# Continuity of care (COC) and amyloid-β PET scan: the CARE-IDEAS study

**DOI:** 10.1186/s13195-022-01126-0

**Published:** 2023-01-07

**Authors:** Emily C. O’Brien, Cassie B. Ford, Corinna Sorenson, Eric Jutkowitz, Megan Shepherd-Banigan, Courtney Van Houtven

**Affiliations:** 1 Department of Population Health Sciences, School of Medicine, Duke University, Durham, NC USA; 2 Duke Clinical Research Institute, School of Medicine, Duke University, Durham, NC USA; 3 School of Public Health, Brown University, Providence, RI USA; 4 Center for Health Services Research in Primary Care, Durham Veterans Affairs Medical Center, Durham, NC USA

**Keywords:** Mild cognitive impairment, care continuity, amyloid-β PET scan, care team communication

## Abstract

**Background:**

High continuity of care (COC) is associated with better clinical outcomes among older adults. The impact of amyloid-β PET scan on COC among adults with mild cognitive impairment (MCI) or dementia of uncertain etiology is unknown.

**Methods:**

We linked data from the CARE-IDEAS study, which assessed the impact of amyloid-β PET scans on outcomes in Medicare beneficiaries with MCI or dementia of uncertain etiology and their care partners, to Medicare claims (2015–2018). We calculated a participant-level COC index using the Bice-Boxerman formula and claims from all ambulatory evaluation and management visits during the year prior to and following the amyloid-β PET scan. We compared baseline characteristics by scan result (elevated or non-elevated) using standardized differences. To evaluate changes in COC, we used multiple regression models adjusting for sociodemographics, cognitive function, general health status, and the Charlson Comorbidity Index.

**Results:**

Among the 1171 cohort members included in our analytic population, the mean age (SD) was 75.2 (5.4) years, 61.5% were male and 93.9% were non-Hispanic white. Over two-thirds (68.1%) had an elevated amyloid-β PET scan. Mean COC for all patients was 0.154 (SD = 0.102; range = 0–0.73) prior to the scan and 0.158 (SD = 0.105; range = 0–1.0) in the year following the scan. Following the scan, the mean COC index score increased (95% CI) by 0.005 (−0.008, 0.019) points more for elevated relative to not elevated scan recipients, but this change was not statistically significant. There was no association between scan result (elevated vs. not elevated) or any other patient covariates and changes in COC score after the scan.

**Conclusion:**

COC did not meaningfully change following receipt of amyloid-β PET scan in a population of Medicare beneficiaries with MCI or dementia of uncertain etiology. Future work examining how care continuity varies across marginalized populations with cognitive impairment is needed.

## Background

Continuity of care (COC) is a multidimensional construct representing the process by which the patient and healthcare team are cooperatively involved over time in healthcare management, with the shared goal of high-quality, cost-effective care [1, 2]. High care continuity is associated with greater patient satisfaction [3–5], improved care team communication, and lower overuse of procedures [6]. With up to 80% of medical errors related to miscommunication arising during care transitions [7], care continuity may influence not only the patient experience, but also risk of adverse clinical events. Results from clinical trials [8] and observational studies [9] suggest that higher care continuity is associated with improved outcomes, and that this association may be particularly important in older adult populations [10, 11]. Associations appear to persist for observed continuity in both primary care and specialty care settings, each of which has been shown to be associated with lower risk of inpatient and ED visits [12].

Given that patients with dementia typically have a high comorbidity burden and often receive care from multiple providers in the ambulatory setting [13], COC may be especially important for reducing unnecessary utilization and improving outcomes. Receiving results of an amyloid-β PET scan could theoretically impact diagnostic uncertainty, subsequent care-seeking patterns and, as a result, care continuity for people with MCI. However no studies to date have examined changes in COC following amyloid-β PET scan. . Additionally, care partners for persons with dementia often play an important role in clinical decision-making and care-seeking behavior. However, whether care partner perception of care involvement meaningfully influences COC has not been well-defined. We evaluated associations between amyloid-β PET scan results (elevated amyloid plaque levels can be indicative of Alzheimer’s disease) and changes in COC from the year prior to the scan to the year following the scan, as well as variation in these associations by care partner-perceived communication with the care team. We hypothesized that COC would change following a receipt of amyloid-β PET scan, with greater changes among those with an elevated scan result due to reductions in care-seeking given the greater certainty of AD diagnosis.

## Methods

### Data sources

Our analysis linked data from two sources. The first data source was the Caregivers' Reactions and Experience, a supplemental study of the Imaging Dementia Evidence for Amyloid Scanning Study (CARE-IDEAS) (www.ideas-study.org). “IDEAS study participants who had a care partner and indicated willingness to be contacted about an additional study were included. Of 3717 IDEAS study participants who agreed to be contacted, 2228 scan recipients and 1872 of their care partners (the “dyad”) completed CARE-IDEAS baseline surveys.” [14] Amyloid scans in the IDEAS study were interpreted based on FDA guidelines by imaging specialists as elevated (cortical tracer retention) or non-elevated (white matter retention only) [15]. The second data source was Medicare inpatient, outpatient, carrier and Master Beneficiary Summary File (MBSF) institutional files for the observation period from 2015 through 2018. For this analysis, we used claims from the one-year period preceding and following each participant’s amyloid-β PET scan date. We excluded participants with the following characteristics: (1) without complete patient-partner dyad baseline survey data; (2) uninterpretable or missing amyloid-β PET scan results; (3) death within one year of the scan; (4) without continuous fee-for-service Medicare coverage in the year prior to and following the scan; and (5) missing information on the care partner-perceived communication with the care team measure, or CAPACITY communication domain. Because COC is less meaningful with fewer healthcare encounters, we additionally excluded participants with fewer than 4 claims for healthcare encounters in either of the 1-year periods before and after the scan.

### Continuity of care

We conceptualized care continuity as dispersion of care across multiple providers, as evidenced by healthcare encounters with distinct clinicians. We used the Bice-Boxerman formula to calculate a participant-level care continuity score based on claims from all ambulatory evaluation and management visits in Medicare claims during the year prior and the year following receipt of the amyloid-β PET scan [16]. The score ranges from 0 to 1, with a score of 0 indicating no continuity of care (all visits to unique providers) and 1 indicating perfect continuity of care (all visits to the same provider). Visits on the date of the scan (+/− 1 day) were excluded from the COC calculation because they are related to the study rather than usual care.

### Statistical analysis

We compared baseline characteristics by scan result using standardized differences for means and proportions. To evaluate changes in COC before and after the scan, we used multiple regression models adjusting for participant age, sex, race, education, cognitive function (TICS-M), general health status, the Charlson Comorbidity Index, and included an indicator for scan result (elevated vs. not elevated amyloid plaque level). Elevated amyloid plaque levels can be indicative of Alzheimer’s disease [17]. To evaluate whether associations changed with caregiver-perceived communication with the care team, we used data from the communication domain of CAPACITY, a validated measure of caregiver-perceived quality of communication with the health care team [18]. We assessed for an interaction between caregiver-perceived communication and scan result on care continuity by including an interaction term for the communication domain score and elevated vs. not elevated scan result in the regression model. Missing covariate data were addressed using a missing category for categorical variables and simple imputation to the mean for continuous variables. Analyses were performed in SAS v9.4. The CARE-IDEAS study was approved by the Brown University Institutional Review Board (#1606001534).

## Results

There were 1171 cohort members included in our analytic population. The mean age (SD) was 75.2 (5.4) years, 61.5% of cohort members were male and 93.9% were non-Hispanic white (Table 1). Over two-thirds of cohort members (68.1%) had an elevated amyloid-β PET scan. Compared with those without an elevated scan, patients with an elevated scan were less likely to be college-educated and had a lower mean TICS-M score (19.7 vs. 22.5). Those with an elevated scan were also less likely to have hypertension, active depression, or be current/former tobacco users than those with a not elevated scan. Mean Charlson Comorbidity Index scores were slightly lower among cohort members with an elevated scan than those with a non-elevated scan (2.66 vs. 3.09). Care partner characteristics, including general health status and CAPACITY communication domain score, were comparable among those with elevated and not elevated scan results (Table 2).

**Table 1:** Study participant characteristics (baseline survey data)

Variable	Overall	Elevated	Not elevated	***p***-value
*N*	1171	798	373	
Continuity of care change				
Pre-scan, mean (SD)	0.15 (0.10)	0.16 (0.10)	0.15 (0.10)	.38
Post-scan, mean (SD)	0.16 (0.11)	0.16 (0.10)	0.15 (0.11)	.07
Change score, mean (SD)	0.00 (0.11)	0.00 (0.11)	-0.00 (0.11)	.23
Patient characteristics				
Age, mean (SD)	75.17 (5.35)	75.60 (5.30)	74.27 (5.35)	< .001
Male	720 (61.5%)	480 (60.2%)	240 (64.3%)	.17
Non-Hispanic white	1099 (93.9%)	750 (94.0%)	349 (93.6%)	.78
Education				.11
High school graduate or less	176 (15.0%)	131 (16.4%)	45 (12.1%)	
Some college	297 (25.4%)	190 (23.8%)	107 (28.7%)	
Bachelor’s degree	292 (24.9%)	204 (25.6%)	88 (23.6%)	
Graduate degree	406 (34.7%)	273 (34.2%)	133 (35.7%)	
Bachelors degree or higher	698 (59.6%)	477 (59.8%)	221 (59.2%)	.86
Cognitive functioning (TICS-M), Mean (SD)	20.59 (6.12)	19.72 (6.19)	22.47 (5.54)	< .001
Patient medical history				
Chronic kidney disease	37 (3.2%)	24 (3.0%)	13 (3.5%)	.66
Hypertension	611 (52.2%)	397 (49.7%)	214 (57.4%)	.01
Atrial fibrillation	114 (9.7%)	74 (9.3%)	40 (10.7%)	.44
Ischemic heart disease	131 (11.2%)	93 (11.7%)	38 (10.2%)	.46
Chronic obstructive pulmonary disease	33 (2.8%)	18 (2.3%)	15 (4.0%)	.09
Diabetes	163 (13.9%)	102 (12.8%)	61 (16.4%)	.10
Active depression	221 (18.9%)	136 (17.0%)	85 (22.8%)	.02
Prior history of stroke or TIA	117 (10.0%)	75 (9.4%)	42 (11.3%)	.32
Congestive heart failure	27 (2.3%)	16 (2.0%)	11 (2.9%)	.32
Multiple sclerosis	*	*	*	.13
Parkinsons	20–30 (1.7–2.6%)	14 (1.8%)	*	.30
Traumatic brain injury	66 (5.6%)	45 (5.6%)	21 (5.6%)	.99
Bipolar affective disorder	17 (1.5%)	*	*	.02
History of acute myocardial infarction	49 (4.2%)	31 (3.9%)	18 (4.8%)	.45
Dyslipidemia	554 (47.3%)	371 (46.5%)	183 (49.1%)	.41
Epilepsy or seizure disorder	29 (2.5%)	18 (2.3%)	11 (2.9%)	.48
Patient uses or has used tobacco	220 (18.8%)	136 (17.0%)	84 (22.5%)	.03
Charlson Comorbidity Index, mean (SD)	2.80 (2.32)	2.66 (2.22)	3.09 (2.48)	.01

**Table 2:** Care partner characteristics

Variable	Overall	Elevated	Not elevated	***p***-value
N	1171	798	373	
Care partner characteristics				
Age, mean (SD)	71.04 (9.11)	71.49 (8.82)	70.07 (9.65)	.02
Male	366 (31.3%)	260 (32.6%)	106 (28.4%)	.15
Non-Hispanic white	1099 (93.9%)	755 (94.6%)	344 (92.2%)	.11
Education				.83
High school graduate or less	153 (13.1%)	103 (12.9%)	50 (13.4%)	
Some college	342 (29.2%)	229 (28.7%)	113 (30.3%)	
Bachelor’s degree	316 (27.0%)	214 (26.8%)	102 (27.3%)	
Graduate degree	360 (30.7%)	252 (31.6%)	108 (29.0%)	
Bachelors degree or higher	676 (57.7%)	466 (58.4%)	210 (56.3%)	.50
Working full- or part-time	275 (23.5%)	190 (23.8%)	85 (22.8%)	.70
General health status (self-assessed)				.25
Excellent	191 (16.3%)	143 (17.9%)	48 (12.9%)	
Very good	512 (43.7%)	346 (43.4%)	166 (44.5%)	
Good	324 (27.7%)	211 (26.4%)	113 (30.3%)	
Fair	112 (9.6%)	76 (9.5%)	30-40 (8.0%-10.7%)	
Poor	30–40 (2.5–3.4%)	22 (2.8%)	*	
General health status (self-assessed), mean (SD)	2.39 (0.96)	2.36 (0.97)	2.45 (0.93)	.09
CAPACITY: communication domain score, mean (SD)	3.07 (0.69)	3.09 (0.68)	3.02 (0.71)	.12

Mean COC for all patients was 0.154 (SD = 0.102; range = 0–0.73) prior to the amyloid-β PET scan and 0.158 (SD = 0.105; range = 0–1.0) in the following year. Pre- and post-scan COC ranges were similar among those with elevated and non-elevated scans. Following the scan, the mean COC index score increased (95% CI) by 0.005 (−0.008, 0.019) points more for elevated relative to not elevated scan recipients, but this change was not statistically significant. After the scan, the COC index score declined for 48.0% of study participants and increased for 50.2% of participants. These percentages were comparable among participants with elevated and not elevated scans.

The association between patient and care partner covariates and post-scan changes in COC are displayed in Table 3. Increasing comorbidity burden, as represented by the Charlson Index, was associated with increases in continuity of care post-scan. There was no association between scan result (elevated vs. not elevated) or any other patient covariates and changes in COC score after the scan. Likewise, none of the care partner covariates examined were associated with statistically significant changes in COC post-scan. Inclusion of the CAPACITY communication domain score in regression models did not materially change results, and no interaction between the communication score and scan result was observed.

**Table 3:** Difference in changes in CoC by amyloid-β scan, with and without covariates

	*Mean difference* *(95% CI: L,U)*	*t*	*p*-value
**Unadjusted**			
Amyloid-β scan	0.005 (−0.008, 0.019)	0.75	0.453
**Predictors**	*β*	*95%CI (U,L)*	*p*-value
**Adjusted**			
Constant	0.004517	(−0.04, 0.05)	0.8429
Elevated scan (ref = not elevated)	0.007087	(−0.01, 0.02)	0.3256
**Patient covariates**			
Age	0.000125	(−0.00, 0.00)	0.8570
TICSm	−0.00015	(−0.00, 0.00)	0.7842
Male sex (ref = female)	−0.00926	(−0.04, 0.02)	0.4912
Bachelor’s or higher (ref = less than Bachelor’s)	0.000364	(−0.01, 0.01)	0.9601
Non-Hispanic white (ref = all other)	0.009614	(−0.02, 0.04)	0.4995
Charlson Comorbidity Index	0.003214	(0.00, 0.01)	0.0256
**Care partner covariates**			
Age	−0.00036	(−0.00, 0.00)	0.4508
Male sex (ref=female)	−0.00242	(−0.03, 0.03)	0.8685
Bachelor’s or higher (ref=less than Bachelor’s)	0.006624	(−0.01, 0.02)	0.3481
Non-Hispanic white (ref = all other)	−0.02064	(−0.05, 0.01)	0.1467
Working, full- or part-time (ref = not working)	−0.00787	(−0.03, 0.01)	0.3721
Care partner general health (ref = excellent)			
Very good	0.01170	(−0.01, 0.03)	0.2156
Good	0.009190	(−0.01, 0.03)	0.3709
Fair	0.005612	(−0.02, 0.03)	0.6751
Poor	0.007673	(−0.03, 0.05)	0.7200

## Discussion

In this study, we linked Medicare claims data to the CARE-IDEAS cohort to evaluate changes in care continuity before and after amyloid-β PET scan and examined whether COC changes varied for people with elevated vs. not elevated scan results and for those with better caregiver-health care team communication. Our three main findings are as follows: (1) mean care continuity overall increased slightly following the scan, but this change was not statistically significant; (2) pre-post changes in care continuity were comparable for those with elevated and not elevated scan results; and (3) caregiver communication with the care team did not appear to modify pre-post changes in care continuity.

Prior studies have demonstrated that low care continuity adversely impacts clinical outcomes and contributes to unnecessary utilization [10, 19]. Among other factors, variation in care continuity is driven by access, care-seeking behaviors, and communication between care providers [20, 21]. We hypothesized that care continuity would change following a receipt of amyloid-β PET scan, with greater changes among those with an elevated scan result due to reductions in care-seeking given the greater certainty of AD diagnosis. While we found improvement in overall care continuity post-scan, this association was not significant, and did not differ by scan result or caregiver-perceived communication. The lack of observed associations between scan result, caregiver-perceived communication and care continuity may be due to several factors. First, while we used a 1-year CoC assessment period to characterize care patterns while maximizing inclusion of study participants, patterns of CoC may differ over longer assessment periods. Second, in this cohort of Medicare beneficiaries, healthcare access may be more equitable than in other cohorts, resulting in more established care patterns that is unlikely to be impacted by the results of any single diagnostic test or procedure. Finally, while caregiver communication with the care team represents one dimension of information flow, other dimensions, such as communication across provider groups for a complex patient population such as the CARE-IDEAS cohort, may be more impactful but were not available in our dataset.

Our study has several limitations worth noting. First, our study population comprises a subsample of a larger population of research study volunteers, the majority of whom are white and college-educated. Given that care fragmentation may disproportionately affect people with lower socioeconomic strata, results may not generalize to populations most likely to be negatively impacted by lack of coordination [22]. Second, the Bice-Boxerman COC score is estimated from dispersion of visits across providers and may not fully capture other important aspects of continuity, like direct provider-to-provider communication [5], or other important aspects of care quality, like adherence to clinical guidelines. Therefore, even a perfect COC score may not translate to ideal care for a given individual. Third, we chose a 1-year period before and after the scan to ensure temporal proximity of COC changes and limit selection bias associated with longer observation time requirements. However, changes in care continuity following scan results may require additional time to become observable. Additionally, we excluded participants who died during the follow-up period, a population who may have had different patterns of care continuity prior to death. Finally, while we were able to adjust for a detailed list of confounders using baseline data from IDEAS, our estimates may be influenced by residual confounding.

There is currently no consensus on the ideal level of care continuity as measured by healthcare encounters in patients with cognitive impairment. Future work is needed to understand how continuity varies across populations with MCI or dementia, and whether this variation is associated with access to and utilization of appropriate care.

## Conclusions

Continuity of care did not meaningfully change following receipt of amyloid β PET scan in a population of Medicare beneficiaries with MCI or dementia of uncertain etiology. Future work examining how care continuity varies across marginalized populations with cognitive impairment is needed.

## Data Availability

The data that support the findings of this study are available in the Brown Digital Repository at https://repository.library.brown.edu/studio/about/, which includes a CARE-IDEAS codebook, and a PDF file with a description of the software used and syntax used for data cleaning and the final analytical models.

## Abbreviations

COC: Continuity of care
PET: Positron emission tomography
CAPACITY: Caregiver Perceptions About Communication With Clinical Team Members
SD: Standard deviation

## References

[1] American Academy of Family Physicians. Continuity of care. Definition of the American Academy of Family Physicians. Available: https://www.aafp.org/about/policies/all/definition-care.html. (Accessed 30 April 2019).

[2] Adler R, Vasiliadis A, Bickell N (2010). The relationship between continuity and patient satisfaction: a systematic review. Fam Pract.

[3] Fan VS, Burman M, McDonell MB, Fihn SD (2005). Continuity of care and other determinants of patient satisfaction with primary care. J Gen Intern Med.

[4] Morgan ED, Pasquarella M, Holman JR (2004). Continuity of care and patient satisfaction in a family practice clinic. J Am Board Fam Pract.

[5] Saultz JW, Lochner J (2005). Interpersonal continuity of care and care outcomes: a critical review. Ann Fam Med.

[6] Romano MJ, Segal JB, Pollack CE (2015). The Association Between Continuity of Care and the Overuse of Medical Procedures. JAMA Intern Med.

[7] Solet DJ, Norvell JM, Rutan GH, Frankel RM (2005). Lost in translation: challenges and opportunities in physician-to-physician communication during patient handoffs. Acad Med.

[8] Wasson JH, Sauvigne AE, Mogielnicki RP (1984). Continuity of outpatient medical care in elderly men. A randomized trial. JAMA..

[9] Parchman ML, Pugh JA, Noel PH, Larme AC (2002). Continuity of care, self-management behaviors, and glucose control in patients with type 2 diabetes. Med Care.

[10] Hussey PS, Schneider EC, Rudin RS, Fox DS, Lai J, Pollack CE (2014). Continuity and the costs of care for chronic disease. JAMA Intern Med.

[11] Wolinsky FD, Bentler SE, Liu L (2010). Continuity of care with a primary care physician and mortality in older adults. J Gerontol A Biol Sci Med Sci.

[12] Bayliss EA, Ellis JL, Shoup JA, Zeng C, McQuillan DB, Steiner JF (2015). Effect of continuity of care on hospital utilization for seniors with multiple medical conditions in an integrated health care system. Ann Fam Med.

[13] Nelis SM, Wu YT, Matthews FE, et al. The impact of comorbidity on the quality of life of people with dementia: findings from the IDEAL study. Age Ageing. 2018. 10.1093/ageing/afy155. 10.1093/ageing/afy155 PMC6503940 30403771

[14] James HJ, Van Houtven CH, Lippmann S (2020). How Accurately Do Patients and Their Care Partners Report Results of Amyloid-beta PET Scans for Alzheimer's Disease Assessment?. J Alzheimers Dis.

[15] Rabinovici GD, Gatsonis C, Apgar C (2019). Association of Amyloid Positron Emission Tomography With Subsequent Change in Clinical Management Among Medicare Beneficiaries With Mild Cognitive Impairment or Dementia. JAMA.

[16] Amjad H, Carmichael D, Austin AM, Chang CH, Bynum JP (2016). Continuity of Care and Health Care Utilization in Older Adults With Dementia in Fee-for-Service Medicare. JAMA Intern Med.

[17] Hardy JA, Higgins GA (1992). Alzheimer's disease: the amyloid cascade hypothesis. Science.

[18] Van Houtven CH, Miller KEM, O'Brien EC (2019). Development and Initial Validation of the Caregiver Perceptions About Communication With Clinical Team Members (CAPACITY) Measure. Med Care Res Rev.

[19] Frandsen BR, Joynt KE, Rebitzer JB, Jha AK (2015). Care fragmentation, quality, and costs among chronically ill patients. Am J Manag Care.

[20] Nelson K, Sun H, Dolan E (2014). Elements of the patient-centered medical home associated with health outcomes among veterans: the role of primary care continuity, expanded access, and care coordination. J Ambul Care Manage.

[21] Price M, Lau FY (2013). Provider connectedness and communication patterns: extending continuity of care in the context of the circle of care. BMC Health Serv Res.

[22] Schrag D, Xu F, Hanger M, Elkin E, Bickell NA, Bach PB (2006). Fragmentation of care for frequently hospitalized urban residents. Med Care.

